# Is 5 mm MMLC suitable for VMAT‐based lung SBRT? A dosimetric comparison with 2.5 mm HDMLC using RTOG‐0813 treatment planning criteria for both conventional and high‐dose flattening filter‐free photon beams

**DOI:** 10.1120/jacmp.v16i4.5415

**Published:** 2015-07-08

**Authors:** Shanmuga V. Subramanian, Vellaiyan Subramani, Shanmugam Thirumalai Swamy, Arun Gandhi, Srinivas Chilukuri, Murugesan Kathirvel

**Affiliations:** ^1^ Research and Development Centre Bharathiar University Coimbatore India; ^2^ Department of Radiation Oncology Yashoda Hospital Hyderabad India; ^3^ Department of Radiation Oncology All India Institute of Medical Sciences New Delhi India

**Keywords:** SBRT, VMAT, FFF, MMLC, HDMLC, RTOG‐0813

## Abstract

The aim of this study is to assess the suitability of 5 mm millennium multileaf collimator (MMLC) for volumetric‐modulated arc therapy (VMAT)‐based lung stereotactic body radiotherapy (SBRT). Thirty lung SBRT patient treatment plans along with their planning target volumes (ranging from 2.01 cc to 150.11 cc) were transferred to an inhomogeneous lung phantom and retrospectively planned using VMAT technique, along with the high definition multileaf collimator (HDMLC) and MMLC systems. The plans were evaluated using Radiation Therapy Oncology Group (RTOG‐0813) treatment planning criteria for target coverage, normal tissue sparing, and treatment efficiency for both the MMLC and HDMLC systems using flat and flattening filter‐free (FFF) photon beams. Irrespective of the target volumes, both the MLC systems were able to satisfy the RTOG‐0813 treatment planning criteria without having any major deviation. Dose conformity was marginally better with HDMLC. The average conformity index (CI) value was found to be 1.069±0.034 and 1.075±0.0380 for HDMLC and MMLC plans, respectively. For the 6 MV FFF beams, the plan was slightly more conformal, with the average CI values of 1.063±0.029 and 1.073±0.033 for the HDMLC and MMLC plans, respectively. The high dose spillage was the maximum for 2 cc volume set (3% for HDMLC and 3.1% for MMLC). In the case of low dose spillage, both the MLCs were within the protocol of no deviation, except for the 2 cc volume set. The results from this study revealed that VMAT‐based lung SBRT using 5 mm MMLC satisfies the RTOG‐0813 treatment planning criteria for the studied target size and shapes.

PACS numbers: 87.53.Ly, 87.53D, 87.56.jk

## I. INTRODUCTION

Stereotactic body radiotherapy (SBRT) with promising results and excellent tumor control rates, as well as limited toxicities to the normal tissues, has become a primary modality of treatment for the medically inoperable early stage lung cancer.^(1)^ SBRT involves precise delivery of few fractions of high dose of radiations to accurately delineated malignant targets. It achieves highly conformal dose distribution, avoiding radiation to normal tissues, during the planning process by providing sharp fall‐off dose gradients outside the target volume.^(2)^ Multileaf collimator (MLC) leaf width plays a major role in creating a highly conformal dose distribution with the steeper dose gradient outside the target. Many studies have reported the impact of MLC leaf width on the stereotactic radiosurgery (SRS) plans.^3^, ^4^, ^5^, ^6^, ^7^, ^8^ Dhabaan et al.^(9)^ compared the 3 mm leaf width BrainLAB micro‐MLC with the 5 mm leaf width Varian Millennium 120 MLC, using the dynamic conformal arc radiotherapy (DCRT) technique and reported that the micro‐MLC had comparatively better conformity and greater normal tissue sparing. Tanyi et al.^(10)^ compared 2.5 mm 120 leaf Varian high‐definition MLC (HDMLC) with the widely used 5 mm 120 leaf millennium MLC (MMLC) using intensity‐modulated radiotherapy (IMRT) and dynamic conformal arc techniques. They reported that the HDMLC has a dosimetric advantage over the MMLC. However, none of the above studies used volumetric‐modulated arc therapy (VMAT) to compare the role of the leaf widths in lung SBRT.

VMAT is another form of IMRT, which allows irradiation with simultaneously changing gantry position, dose rate, and MLC. Several studies have shown the potential benefit of VMAT technique over IMRT in reducing the treatment time without compromising the planned quality in the treatment of lung cancers.^11^, ^12^ Jiang et al.^(13)^ compared IMRT with single arc/partial arc VMAT for locally advanced lung cancer and concluded that the VMAT plans achieves better conformal distribution with lower $V_{20}$ and mean lung dose to total lung, as well as the contralateral lung, with reduced treatment time. The new generation of linear accelerators used in radiotherapy is designed without using flattening filter to produce unflattened photon beam and is, thus, referred to as flattening filter‐free (FFF) linear accelerator. Since flattening filter is not used in the photon beam, the dose rate can be increased by two to four times of the conventional treatment so that the delivery time can be faster. Reggiori et al.^(14)^ have reported considerable reductions in the treatment time, along with some improvement in healthy sparing tissue, by using the high‐dose rate FFF beams in VMAT‐based lung SBRT.

All the previous dosimetric studies showed that the difference in PTV coverage and normal tissue sparing was better with the finer 2.5 mm HDMLC as compared to the 5 mm MMLC. However, institutions across the world use the 5 mm MMLC for stereotactic treatments of lung cancer, as it was found adequate by the report of American Association of Physicists in Medicine Task Group (AAPM TG −101).^(15)^ Hence, the current study explores the suitability of 5 mm MMLC for VMAT‐based SBRT by comparing it with 2.5 mm HDMLC using Radiation Therapy Oncology Group (RTOG‐0813) treatment planning criteria for both conventional 6 MV flat, as well as high‐dose rate 6 MV FFF, photon beams.

## II. MATERIALS AND METHODS

### A. Phantom and patient selection

Out of the 110 SBRT lung patients treated at our institute, only 30 patients were selected for this study on the basis of complexities of the target volume. The patients were grouped based on the target volume such that three patients could be included in each group. Further, each group was subclassified, based on the shape of the target (simple‐spherical, moderate‐ellipsoid, and complex‐c‐shaped targets). For example, as shown in Table 1, set 1 has three patients with target volume of 2.01, 2.39, and 2.8 cc, respectively. The 2.01 cc volume falls under the simple target category, 2.39 cc volume falls under the moderate target, and the 2.8 cc is under the complex‐shaped target. Additionally, it was ensured that, for the entire range of volumes, three patients planning target volumes were selected from each volume to represent the simple, moderate, and complex shapes. This was done in order to know the effect of the leaf width in diverse target shape settings.

**Figure 1:**
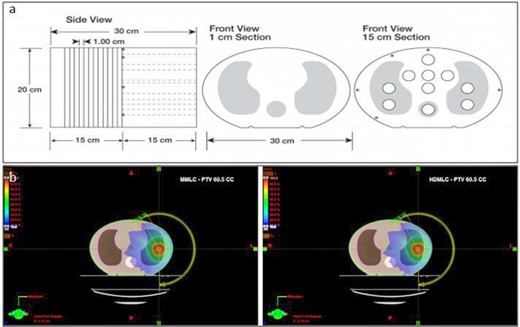
CIRS‐IMRT thorax phantom (a) and (b) typical dose distribution in CIRS‐IMRT thorax phantom for PTV 60.5 cc, comparing MMLC and HDMLC plans.

All the target volumes, along with their respective treatment plans, were transferred into an inhomogeneous lung phantom (CIRS‐IMRT Thorax phantom‐model 002LFC; CIRS, Norfolk, VA) so as to confine and extract the volume dependence information without introducing any biases through the anatomical variability of the patients. The inhomogeneous lung phantom (Fig. 1) basically consists of a 15 cm thick slice and 15 standard slices (each 1 cm thick). They are composed of water‐equivalent materials with inhomogeneities mimicking lung (density, $0.21\;g/{\text{cm}}^{3}$) and bone vertebra (density, $1.6\;g/{\text{cm}}^{3}$) with different geometry and density in the longitudinal direction. Apart from the body, both lungs and bony vertebra were contoured to know the effect of leaf width on these volumes. All the patients were treated using Varian Clinac‐2100CD (Varian Medical System, Palo Alto, CA), which is equipped with 5 mm MMLC; hence, for each target volume, 5 mm MMLC plans were considered as the reference plans for the study.

**Table 1:** Thirty lung SBRT patient planning target volumes based on target shape and volume

*Patients Group*	*PTV Volume (cc)*			
	*Simple*	*Moderate*	*Complex*	*Average*
Set 1	2.01	2.39	2.80	2.39
Set 2	5.21	4.98	5.50	5.23
Set 3	10.51	10.97	9.94	10.47
Set 4	20.7	20.47	19.37	20.18
Set 5	39.05	40.31	41.02	40.13
Set 6	60.88	60.50	59.22	60.20
Set 7	79.27	81.71	80.84	80.60
Set 8	99.85	100.76	100.86	100.49
Set 9	120.47	119.02	120.00	119.83
Set 10	148.07	149.52	150.11	149.23

### B. Treatment planning

All treatment plans were performed with the Eclipse treatment planning system Version ‐ 11 (Varian Medical System) to exclude any bias due to the effect of different planning algorithms. All patient plans with their respective target volumes were transferred to the phantom with identical gantry and collimator positions. The original patient plans along with the patient optimization parameters were also transferred to the phantom. Four arcs (two coplanar and two noncoplanar) as used in patient plans were reoptimized with 5 mm MMLC using the VMAT (RapidArc; Varian Medical Systems) technique. All VMAT plans were planed using 6 MV flat beam (maximum dose rate of 600 MU/min) for each volume in the inhomogeneous lung phantom. Treatment planning was performed to achieve the acceptable dose distributions based on the RTOG‐0813 dose constraints criteria. Comparative plans were generated from the corresponding 5 mm MMLC RapidArc plans for each volume by reoptimizing with 2.5 mm HDMLC system. Dose calculation was performed with Acuros XB advanced dose calculation algorithm.^(16)^ Accuracy of Acuros XB advanced dose calculation algorithm was comprehensively studied by Han et al.^(17)^ in both homogenous and heterogeneous media. For the studied SBRT lung patients, the difference between Acuros and collapsed cone convolution (CCC) algorithm dose calculation accuracy is less than 1%, which correlates well with the study performed by Kathirvel et al.^(16)^ The dose prescription of 60 Gy in 5 fractions with 12 Gy per fraction was used as a standard for both the MMLC and HDMLC treatment plans. All plans were computed such that the prescribed dose encompassed 95% of the PTV volume, with 99% of the target volume receiving a minimum of 90% of the prescribed dose. The Clinac‐2100CD was recently upgraded with 6 MV FFF beam; thus, we could explore the influence of the respective MLC system in FFF beam, as well. In order to analyze the effect of leaf width on FFF beam, all the plans with both the MLC systems were reoptimized and replanned using 6 MV FFF beams (maximum dose rate of 1400 MU/min) with identical optimization and dose calculation parameters.

### C. Evaluation of treatment plans

RTOG‐0813 dose constraints for treatment planning were used to evaluate both the MMLC and HDMLC plans. Per the RTOG‐0813 treatment planning guidelines, a successful treatment plan should satisfy the following criteria.

#### C.1 Prescription isodose surface coverage

The prescription isodose surface should be chosen such that 95% of the target volume (PTV) is conformably covered by the prescription isodose surface and 99% of the target volume (PTV) receives a minimum of 90% of the prescription dose.

#### C.2 Target dose heterogeneity

The prescription isodose surface selected must be ≥60% of the dose at the center of mass of the PTV and ≤90% of the dose at the center of mass of the PTV (COMPTV). The COMPTV corresponds to the normalization point (100%).

#### C.3 High dose spillage

##### C.3.1 Location

Any dose >105% of the prescription dose should occur primarily within the PTV itself and not within the normal tissues outside the PTV. Therefore, the cumulative volume of all the tissue outside the PTV receiving a dose >105% of the prescription dose should be no more than 15% of the PTV volume.

##### C.3.2 Volume

Conformality of PTV (CI) coverage should be judged in such a way that the ratio of the volume of prescription isodose to the volume of PTV is ideally <1.2 (Table 2).

**Table 2:** RTOG‐0813 treatment planning protocol: conformality of prescribed dose for calculations based on deposition of photon beam energy in heterogeneous tissue

	*Ratio of Prescription Isodose Volume to the PTV Volume*		*Ratio of 50% Prescription Isodose Volume to the PTV Volume,* $R_{50\%}$		*Maximum Dose (in % of dose prescribed) @ 2 cm from PTV in Any Direction, D2 cm*		*Percent of Lung Receiving 20 Gy Total or More,* $V_{20\text{Gy}}\;(\%)$	
*PTV Volume (cc)*	*Deviation*		*Deviation*		*Deviation*		*Deviation*	
	*None*	*Minor*	*None*	*Minor*	*None*	*Minor*	*None*	*Minor*
1.8	<1.2	<1.5	<5.9	<7.5	<50.0	<57.0	<10	<15
3.8	<1.2	<1.5	<5.5	<6.5	<50.0	<57.0	<10	<15
7.4	<1.2	<1.5	<5.1	<6.0	<50.0	<58.0	<10	<15
13.2	<1.2	<1.5	<4.7	<5.8	<50.0	<58.0	<10	<15
22.0	<1.2	<1.5	<4.5	<5.5	<54.0	<63.0	<10	<15
34.0	<1.2	<1.5	<4.3	<5.3	<58.0	<68.0	<10	<15
50.0	<1.2	<1.5	<4.0	<5.0	<62.0	<77.0	<10	<15
70.0	<1.2	<1.5	<3.5	<4.8	<66.0	<86.0	<10	<15
95.0	<1.2	<1.5	<3.3	<4.4	<70.0	<89.0	<10	<15
126.0	<1.2	<1.5	<3.1	<4.0	<73.0	>91.0	<10	<15
163.0	<1.2	<1.5	<2.9	<3.7	<77.0	>94.0	<10	<15

#### C.4 Low dose spillage

The falloff gradient beyond the PTV extending into normal tissue structures must be rapid in all the directions and meet the following criteria.

##### C.4.1 Location

The maximum total dose over all fractions in Gray (Gy) to any point 2 cm or greater away from the PTV in any direction must not be greater than D2cm (D2cm is specified in the Table 2).

##### C.4.2 Volume

The ratio of the volume of 50% of the prescription dose isodose to the volume of PTV must not be greater than $R_{50\%}$ ($R_{50\%}$ is specified in Table 2).

#### C.5 Critical organ — lung

Apart from the target and normal tissues, the contoured volume of total lung receiving a maximum dose of 12.5 Gy ($V_{12.5\text{Gy}}$) and 13.5 Gy ($V_{13.5\text{Gy}}$) should be kept below 1500 cc and 1000 cc, respectively. For all target volumes, the total volume of lung that receives 20 Gy ($V_{20\text{Gy}}$) has to be kept below 10% (15% for minor deviations) of the prescribed dose.

In addition to the above RTOG criteria, the following parameters were also included in the final evaluation.

#### C.6 Maximum dose

Maximum dose within the body (in terms of the prescribed dose) for both MLC system plans were evaluated for each beam types.

#### C.7 Planning efficiency (MU/Gy)

Planning efficiency, which is defined as the ratio of cumulative sum of monitor units (MUs) per fraction to the dose per fraction, was also calculated for both MLC systems using both beam types.

### D. Gain ratio

It is defined as the ratio that is used to evaluate the improvement in the index between the rival plans (i.e., a plan with a 2.5 mm HDMLC versus a plan with a 5 mm MMLC).^(17)^

(1) $$Gain\;Ratio(\%)=\frac{\left(Index_{2.5\;mm}MLC-Index_{5\;mm}MLC\right)}{Index_{5\;mm}MLC}$$

### E. Statistical analysis

A paired *t*‐test with two‐tailed distribution was used to determine whether the difference between MLC treatment plans were statistically significant. A p‐value <0.05 was considered to be statistically significant.

## III. RESULTS

### A. Prescription isodose surface

In the conventional 6 MV flat beam, the average prescription isodose varied from 79.1% to 84% of the maximum dose for HDMLC and 78.3% to 83% of the maximum dose for MMLC. In the 6 MV FFF beam, the average prescription isodose varied from 79.2% to 84.2% of the maximum dose for HDMLC and 77.7% to 83% of the maximum dose for MMLC.

### B. Target dose heterogeneity

The prescription isodose surface was well within the 60%–90% limit of the RTOG guidelines, as shown in Table 3. For the conventional flat beam, the average PTV minimum dose varied from 94.6% to 96.6% of the prescribed dose for HDMLC and 94.1% to 95.7% of the prescribed dose for MMLC. For the FFF beam, the average PTV minimum dose varied from 95.3% to 96.6% of the prescribed dose for HDMLC and from 94.3% to 96.4% for MMLC of the prescribed dose.

**Table 3:** Evaluation of PTV maximum (Max), minimum (Min), and prescription isodose for 6 MV flat and FFF beam

	*6 MV Flat Beams*						*6 MV FFF Beams*					
*Mean PTV Volume (cc)*	*Max dose (in % of dose prescribed)*		*Min dose (in % of dose prescribed)*		*Prescription Isodose (%)*		*Max dose (in % of dose prescribed)*		*Min dose (in % of dose prescribed)*		*Prescription Isodose (%)*	
	*HD* ^a^	*MM* ^b^	*HD*	*MM*	*HD*	*MM*	*HD*	*MM*	*HD*	*MM*	*HD*	*MM*
2.39	124.8	126.7	96.2	95.2	80.4	79.2	122.8	126.1	96.6	96.4	79.3	79.4
5.23	122.2	128.5	96.2	95.7	82.2	78.3	121.6	124.3	96.0	95.9	81.2	77.7
10.47	118.5	120.3	95.9	94.8	84.0	83.0	118.7	119.7	96.5	95.4	84.2	83.0
20.18	121.1	123.8	96.6	95.3	80.6	80.2	121.1	124.6	96.4	95.7	82.5	80.8
40.12	123.1	123.1	95.4	94.5	81.1	80.7	121.8	123.5	95.3	95.1	81.0	81.2
60.20	122.4	123.5	95.0	94.8	81.5	80.4	122.7	124.6	95.8	95.2	81.6	81.0
80.60	121.8	126.7	95.4	94.1	81.2	79.9	121.7	125.2	95.7	95.1	82.0	79.0
100.49	123.6	124.1	94.6	94.2	80.2	80.8	124.6	123.8	96.3	95.7	81.2	81.9
119.83	124.8	126.4	96.2	95	79.8	80.4	125.9	123.6	96.1	94.3	81.2	80.5
149.23	121.9	122.9	95.6	95.1	79.1	78.8	121.9	126.9	95.7	95.5	79.2	80.0
Mean	122.4	124.6	95.7	94.8	81.0	80.17	122.3	124.2	96.5	95.4	81.3	80.5
STD	1.857	2.42	0.623	0.499	1.37	1.29	1.953	1.929	0.417	0.564	1.45	1.51
p‐value	0.007		0.001		0.068		0.02		0.004		0.090	

a ^a^ HD: 2.5 mm 120 leaf Varian high definition MLC (HDMLC).

b ^b^ MM: 5 mm 120 leaf Varian millennium MLC (MMLC).

### C. High dose spillage (HDS)

#### C.1 Location

For both HDMLC and MMLC plans, the high dose spillage was within the tolerance of 15% of their respective PTV volumes. The HDS outside PTV for all the target volumes (except for 2 cc) was less than 0.5% of the respective PTV volume and, hence, was not tabulated. In case of flat beams, the average high dose spillage for 2 cc set‐1 was 3% and 3.1% of the average PTV volume for HDMLC and MMLC plans, respectively. In the case of FFF beam, the average high dose spillage was 2.9% and 3% of the average PTV volume for HDMLC and MMLC plans, respectively.

#### C.2 Volume

For flattened conventional beams, the average CI value was 1.069±0.034 (±1 SD) and 1.075±0.0380 (±1 SD) for HDMLC and MMLC plans, respectively (Table 4). For FFF beams, the plan was slightly more conformal with the average CI values of 1.063±0.029 and 1.073±0.033 for HDMLC and MMLC plans, respectively (Table 5). No statistical difference was observed between the CI values of the two MLC plans irrespective of the type of beam (p=0.096 for 6 MV flat beam and p=0.063 for 6 MV FFF beam).

**Table 4:** Evaluation of CI, $R_{50\%}$, and MU parameter for 6 MV flat beams using RTOG‐0813 protocol criteria

*Mean PTV Volume (cc)*	*CI*			*RTOG 0813 Deviation Minor*	$R_{50\%}$			*RTOG 0813 Deviation Minor*	*MU/cGy*	
	*HD*	*MM*	*GR*		*HD*	*MMLC*	*GR*		*HD*	*MMLC*
2.39	1.143	1.150	−0.7	1.2−1.5	6.017	6.504	−7.5	5.78−7.2	4.242	4.084
5.23	1.110	1.116	−0.5	1.2−1.5	5.178	5.580	−7.2	5.34−6.3	3.786	3.746
10.47	1.031	1.041	−0.9	1.2−1.5	4.360	4.630	−5.8	4.88−5.89	3.624	3.813
20.18	1.018	1.022	−0.4	1.2−1.5	3.851	4.080	−5.6	4.51−5.56	3.320	3.413
40.12	1.068	1.079	−1.0	1.2−1.5	3.409	3.570	−4.5	4.18−5.18	3.673	3.565
60.20	1.061	1.064	−0.3	1.2−1.5	3.109	3.220	−3.4	3.75−4.9	3.536	3.635
80.60	1.078	1.068	0.9	1.2−1.5	3.050	3.145	−3.0	3.42−4.63	3.663	3.689
100.49	1.070	1.071	−0.1	1.2−1.5	3.114	3.204	−2.8	3.26−4.33	3.544	3.595
119.83	1.063	1.065	−0.2	1.2−1.5	3.041	3.120	−2.5	3.14−4.07	3.576	3.339
149.23	1.064	1.065	−0.1	1.2−1.5	2.840	2.910	−2.4	2.97−3.81	3.492	3.374
Mean	1.069	1.075			3.797	3.996			3.646	3.625
STD	0.034	0.380			1.069	1.215			0.243	0.226
p‐value	0.096				0.002				0.643	

GR=Gain Ratio

**Table 5:** Evaluation of CI, $R_{50\%}$, and MU parameter for 6 MV FFF beams using RTOG‐0813 protocol criteria

*Mean PTV Volume (cc)*	*CI*			*RTOG 0813 Deviation Minor*	$R_{50\%}$			*RTOG 0813 Deviation Minor*	*MU/cGy*	
	*HD*	*MM*	*GR*		*HD*	*MMLC*	*GR*		*HD*	*MMLC*
2.39	1.128	1.131	−0.2	1.2−1.5	5.802	6.255	−7.2	5.78−7.2	4.322	4.054
5.23	1.107	1.113	−0.5	1.2−1.5	4.773	5.133	−7.0	5.34−6.3	4.253	4.027
10.47	1.026	1.031	−0.5	1.2−1.5	4.190	4.432	−5.5	4.88−5.89	3.904	3.863
20.18	1.016	1.025	−0.9	1.2−1.5	3.709	3.919	−5.4	4.51−5.56	3.430	3.422
40.12	1.071	1.075	−0.4	1.2−1.5	3.291	3.442	−4.4	4.18−5.18	3.723	3.683
60.20	1.063	1.072	−0.8	1.2−1.5	2.997	3.086	−2.9	3.75−4.9	3.579	3.661
80.60	1.066	1.073	−0.6	1.2−1.5	3.026	3.116	−2.9	3.42−4.63	3.799	3.878
100.49	1.065	1.071	−0.6	1.2−1.5	3.068	3.151	−2.6	3.26−4.33	3.231	3.139
119.83	1.064	1.066	−0.1	1.2−1.5	2.989	3.061	−2.4	3.14−4.07	3.859	3.805
149.23	1.063	1.067	−0.4	1.2−1.5	2.908	2.956	−1.6	2.97−3.81	3.434	3.432
Mean	1.063	1.073			3.675	3.855			3.753	3.696
STD	0.029	0.033			0.969	1.104			0.353	0.292
P value	0.063				0.002				0.148	

GR=Gain Ratio

### D. Low dose spillage (LDS)

#### D.1 Location

The maximum dose (in % of dose prescribed) at 2 cm from PTV in any direction was tabulated along with RTOG‐0813 specifications (6, 7). Both the MLC plans exhibited similar low dose spillage for both the beam types with a subtle difference favoring HDMLC plans, where the average D2cm is 0.61% less in flat beams and 0.87% less in the case of FFF beams, as compared to the MMLC plans. However, these differences were not statistically significant.

**Table 6:** Evaluation of D2 cm and lung $V_{20\text{Gy}}$, $V_{13.5\text{Gy}}$, $V_{12.5\text{Gy}}$, and $V_{5\text{Gy}}$ parameter for 6 MV flat beams

				*Lung*								
*Mean PTV Volume (cc)*	*D2 cm (in % of dose prescribed)*		*RTOG 0813 Criteria for Deviation Minor (%)*	$V_{20Gy}\;(\%)$		*RTOG 0813 Criteria for Deviation Minor (%)*	$V_{13.5Gy}\;(\text{cc})$		$V_{12.5Gy}\;(\text{cc})$		$V_{5Gy}\;(\text{cc})$	
	*HD*	*MM*		*HD*	*MM*		*HD*	*MM*	*HD*	*MM*	*HD*	*MM*
2.39	40.17	42.23	50−57	0.63	0.69	10−15	49.5	55.2	54.8	61.5	159.7	171.1
5.23	43.47	42.57	50−57	1.11	1.14	10−15	79.2	81.6	87.4	90.1	227.7	228.7
10.47	45.83	43.80	50−58	2.22	2.20	10−15	121.4	133.9	133.8	146.7	302.7	327.5
20.18	48.57	50.13	52−58	2.90	3.10	10−15	152.0	161.0	167.2	176.0	397.0	406.8
40.12	51.03	52.97	59−69	4.16	4.00	10−15	244.4	255.7	262.6	273.7	448.1	465.4
60.20	54.93	53.40	64−81	5.22	5.14	10−15	355.2	373.1	375.7	395.7	563.1	582.4
80.60	57.87	56.97	68−88	6.26	6.57	10−15	435.1	456.3	456.1	475.6	728.8	774.6
100.49	63.13	66.03	70−89	8.40	8.30	10−15	530.0	538.1	544.9	555.7	756.7	809.2
119.83	65.07	67.27	72−90	9.20	9.30	10−15	606.1	607.8	626.8	627.5	812.8	848.9
149.23	66.87	67.60	75−92	9.30	9.36	10−15	650.8	658.0	669.2	678.2	1083.8	1102.0
Mean	53.69	54.30		4.94	4.98		322.3	332.1	337.9	348.1	548.0	571.7
STD	9.39	10.01		3.27	3.28		224.7	224.9	228.7	229.3	294.39	304.1
p‐value	0.313			0.395			0.01		0.01		0.01	

**Table 7:** Evaluation of D2 cm and lung $V_{20\text{Gy}}$, $V_{13.5\text{Gy}}$, $V_{12.5\text{Gy}}$, and $V_{5\text{Gy}}$ parameter for 6 MV FFF beams

				*Lung*								
*Mean PTV Volume (cc)*	*D2 cm (in % of dose prescribed)*		*RTOG 0813 Criteria for Deviation Minor (%)*	$V_{20Gy}\;(\%)$		*RTOG 0813 Criteria for Deviation Minor (%)*	$V_{13.5Gy}\;(\text{cc})$		$V_{12.5Gy}\;(\text{cc})$		$V_{5Gy}\;(\text{cc})$	
	*HD*	*MM*		*HD*	*MM*		*HD*	*MM*	*HD*	*MM*	*HD*	*MM*
2.39	40.23	38.07	50−57	0.60	0.66	10−15	48.4	54.7	54.6	61.3	160.1	166.2
5.23	42.60	42.37	50−57	1.03	1.05	10−15	71.1	76.8	78.4	84.8	207.3	219.9
10.47	43.73	45.50	50−58	2.23	2.11	10−15	115.1	125.3	126.4	137.9	284.2	309.9
20.18	48.60	50.30	52−58	3.13	3.16	10−15	147.7	156.8	161.5	170.4	388.3	391.5
40.12	50.97	53.23	59−69	3.64	3.73	10−15	232.7	253.7	251.2	272.6	423.8	446.1
60.20	52.67	53.20	64−81	4.77	4.81	10−15	283.6	356.6	305.3	377.9	483.5	546.9
80.60	58.30	58.67	68−88	6.10	6.12	10−15	431.7	450.2	447.1	466.4	658.5	684.2
100.49	64.10	66.67	70−89	8.26	8.13	10−15	515.2	520.5	531.2	537.2	729.6	746.0
119.83	54.97	57.57	72−90	8.80	9.00	10−15	596.2	606.1	613.6	623.7	780.4	796.0
149.23	65.07	64.40	75−92	9.05	9.30	10−15	633.5	646.6	649.2	663.3	956.9	965.4
Mean	52.120	52.990		4.76	4.80		307.5	324.7	321.9	339.6	507.3	527.2
STD	8.650	9.240		3.16	3.21		221.1	222.4	224.0	225.7	264.8	265.5
P value	0.253			0.253			0.03		0.02		0.01	

#### D.2 Volume

The average value of $R_{50\%}$ along with the gain ratio and p‐values of the paired *t*‐test comparing corresponding planning techniques of MLC plans for both flat and FFF beams are summarized in 4, 5. Unlike CI, the $R_{50\%}$ and gain ratio exhibited lower values with HDMLC with a maximum gain ratio of 7.5% for flat beams and 7.2% for FFF beams. This maximum improvement was found to be with the smallest average target volume of 2.39 cc with a p‐value of 0.002, which was statistically significant. The results indicated that low dose spillage was better with HDMLC plans as compared to the MMLC plans.

### E. Lung volume

6, 7 shows that the $V_{20\text{Gy}}$, $V_{13.5\text{Gy}}$, and $V_{12.5\text{Gy}}$ values for both MLC systems were far below the allowed RTOG criteria for minor deviation. The mean average $V_{20\text{Gy}}$ was marginally better for HDMLC with 0.8% and 0.84% less than MMLC for flat and FFF beams, respectively. For flat beams, the mean average $V_{13.5\text{Gy}}$, $V_{12.5\text{Gy}}$, and $V_{5\text{Gy}}$ for HDMLC and MMLC plans were 322.3±224.68 cc, 337.85±228.72 cc, 548±294.39 cc, and 332.07±224.99 cc, 348.07±229.27 cc, 571.65±304.05 cc, respectively. For FFF beams, the mean average $V_{13.5\text{Gy}}$, $V_{12.5\text{Gy}}$ and $V_{5\text{Gy}}$, for HDMLC and MMLC plans were 307.52±221.08 cc, 321.85±224.02 cc, 507.26±264.79 cc, and 324.73±222.42 cc, 339.55±225.65 cc, 527.21±265.45 cc, respectively.

### F. Maximum dose

Maximum dose point was within the PTV in all the plans irrespective of MLC systems or the beam types. For the conventional flat beam, as shown in Table 3, the average maximum point dose varied from 118.5% to 124.8% of the prescribed dose for HDMLC, whereas it varied from 120.3% to 128.5% of the prescribed dose for MMLC. In the case of FFF beams, the average maximum point dose varied from 118.7% to 125.9% for HDMLC, whereas it varied from 119.7% to 126.9% of the prescribed dose for MMLC.

### G. Treatment efficiency

As shown in the 4, 5, the mean average MU/cGy for HDMLC was slightly higher both for the flat and FFF beams. The difference was more pronounced for the smallest target volumes (mean PTV volume=2.39 cc). However, no statistically significant difference was found between both the MLC plans (p=0.148 for flat beam; p=0.643 for FFF beam).

## IV. DISCUSSION

The linear accelerator‐based SRS is extensively used for treating small extracranial lesions. One of the major advantages of this technique is the ability to conform the dose distribution to the planning target volume PTV, thus, providing a steep dose gradient between PTVs and the normal tissues. This is realized by using dedicated MLC system with smaller leaves. Studies have shown that, with smaller MLC leaves, linear accelerator‐based SRS with multiple static beams allows better normal tissue sparing for irregular shaped targets than the multiple arching beams using the circular collimators.^18^, ^19^ One of the important studies to assess the impact of MLC on dose distribution was performed by Bortfeld et al.^(4)^ They showed that theoretically calculated optimum leaf width for 6 MV photon beam was in the range of 1.5−2.0 mm. The dosimetric benefit of thin leaf MLCs has been investigated for small target using IMRT technique.^5^, ^6^, ^7^, ^8^, ^9^, ^10^ Tanyi et al.^(10)^ analyzed the impact of two MLC systems for linear accelerator‐based intracranial SRS using three‐dimensional conformal radiotherapy (3D CRT), IMRT, and DCRT. They found a small dosimetric advantage of 2.5 mm leaf width MLC system over 5 mm MLC system in terms of dose conformation, normal tissue exposure, and a rapid dose falloff. Wu et al.^(20)^ evaluated 2.5 mm HDMLC versus 5 mm MMLC for three groups of tumor — spine, brain, and liver. They found that 2.5 mm finer leaf width in combination with IMRT could yield dosimetric benefits in radiosurgery and hypofractionated stereotactic radiotherapy (HSRT). Both the studies raised important questions regarding the significance of small dosimetric benefit with 2.5 mm MLC over 5 mm MLC. Recently, Chae et al.^(21)^ studied the effect of MLC width on radiosurgery planning for spine lesion treatments using the IMRT and VMAT techniques. They concluded that, although smaller (2.5 mm) MLC leaf width provides improved target coverage, the effect of MLC width seems to be more in IMRT than in VMAT.

In our study, the suitability of 5 mm MLC was investigated by comparing the 2.5 mm HDMLC using the RTOG‐0813 treatment planning criteria. In case of PTV, especially in CI, no significant differences were observed between the two collimating systems irrespective of the type of beams. The 2.5 mm HDMLC plans were presented with some benefit in terms of maximum and minimum doses to PTV. However, the differences were relatively smaller and both MLC plan values were below the RTOG criteria for minor deviation. In terms of normal tissue toxicity, except for 2.39 cc, both MLC plans satisfied the RTOG criteria for high and low dose spillage. Chae et al.^(21)^ showed that, for smaller and complex target volumes, the gradient index (GI) was less statistically significant with IMRT whereas the difference was marginal with VMAT. We also found similar results with the high dose and low dose spillage, and the average 2.39 cc target volume for HDMLC plans were found to be marginally better than the 5 mm MMLC plans.

For the target volumes other than 2.39 cc, both the MLC system plans were able to satisfy the RTOG criteria without any deviation. On the other hand, results with $R_{50\%}$, $V_{13.5\text{Gy}}$, $V_{12.5\text{Gy}}$, and $V_{5\text{Gy}}$ comparative indices showed statistically significant reduction favoring HDMLC over MMLC. Comprehensively, it was observed that the results were dependent on the tumor size and shape, and that both target coverage and normal tissue toxicity favored the HDMLC plans. Although statistically significant differences were observed, the observed differences in absolute terms were quite small and might have minute or no clinical significance. No significant difference was observed in terms of treatment efficiency with MU/Gy. MU/Gy was marginally higher with HDMLC plans for smaller targets because of the large number of smaller segments as compared with the MMLC plans.

**Figure 2:**
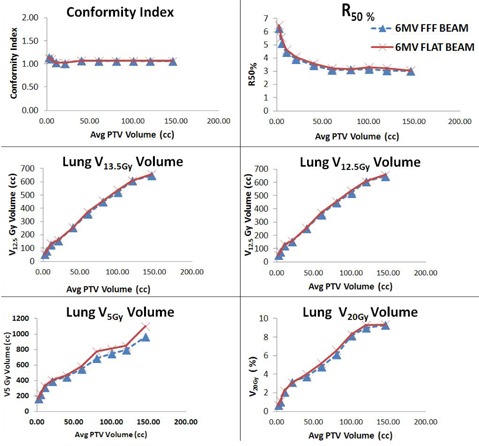
Comparison of treatment planning indices of MMLC (5 mm MLC) for both 6 MV flat and 6 MV FFF beams.

The 6 MV FFF beams exhibited a marginally better CI for both the MLC systems as compared to the flat beams. The plot (see Fig. 2) of treatment planning indices of MMLC comparing 6 MV flat and 6 MV FFF beams showed a better normal tissue sparing that favored 6 MV FFF beam. The plot of treatment planning indices, except for CI, showed that statistically significant differences were obtained favoring 6 MV FFF beam for all other indices such as with $R_{50\%}$, $V_{20\text{Gy}}$, $V_{13.5\text{Gy}}$, $V_{12.5\text{Gy}}$, and $V_{5\text{Gy}}$. The reduction in normal tissue toxicity indices was attributed to the lower energy spectra of the Varian 6 MV FFF beam. The quality index (QI) of 6 MV flat and 6 MV FFF beams was found to be 0.6659 and 0.6291, respectively. In addition, the MLC transmission and dosimetric leaf gap for 6 MV FFF beam were 1% and 1.1 mm as compared to 1.5% and 2 mm for 6 MV flat beam, respectively. Moreover, the comparison of penumbra of 6 MV flat beam with 6 MV FFF beam for MMLC in Table 8 revealed the softer photon beam spectrum and missing scatter from flattening filter resulting in a smaller penumbra in 6 MV FFF beam.^(22)^ All the above parameters (MLC transmission, dosimetric leaf gap, lower energy spectra, and penumbra) had a positive impact on reducing the $V_{20\text{Gy}}$, $V_{13.5\text{Gy}}$, $V_{12.5\text{Gy}}$, and $V_{5\text{Gy}}$ with 6 MV FFF beam.

The current study is purely a treatment planning study on a single treatment planning system with no dosimetric verification. Dosimetric verification was not possible as we have used the same beam parameters of a single machine with identical beam configuration, dose constraints, and optimization parameters for both the MLC systems. However, the difference observed in this study is not only because of the MLC leaf width, but also due to other factors such as leaf transmission, leakage, tongue and groove, and source to MLC distance. In addition, during the actual patient treatment, the MLC interplay effect due to the tumor motion and the calculation accuracy of the model‐based algorithms in lung medium needs to be accounted before taking the final decision of treating the lung SBRT patients with 5 mm MMLC.

**Table 8:** Comparison of penumbra of 6 MV flat beam with 6 MV FFF beam for MMLC

		P_20‐80 (mm)_	
Field Size	Depth (cm)	6 MV Flat Beam	6 MV FFF Beam^a^
3×3	1.5	3.5	3.2
3×3	10	4.6	4.1
5×5	1.5	3.8	3.4
5×5	10	4.5	3.9

a ^a^ Measured using normalization method based on the inflection points.

## V. CONCLUSIONS

The results of this study revealed that VMAT‐based SBRT using 5 mm MMLC satisfies the RTOG‐0813 treatment planning criteria for the studied target size and shapes. Additionally, the introduction of FFF beams, which has softer energy spectra, lower MLC transmission, and lesser penumbra, helps in further reducing the normal tissue dose indices, as compared to the conventional flat beams, for both the MLC systems.
